# Preparation and Characterization of Polysaccharide-Based Hydrogels for Cutaneous Wound Healing

**DOI:** 10.3390/polym14091716

**Published:** 2022-04-22

**Authors:** Hongyan Xue, Meng Sun, Xiaoliang Zhao, Yonggang Wang, Jinxin Yan, Weijie Zhang

**Affiliations:** School of Life Science and Engineering, Lanzhou University of Technology, Lanzhou 730050, China; xuehongyan926@163.com (H.X.); sunmeng2541016286@163.com (M.S.); zhxl819@163.com (X.Z.); wangyg@lut.cn (Y.W.); yanjinxin8210@163.com (J.Y.)

**Keywords:** hydrogel, chitosan, *Lonicera japonica* Thunb. polysaccharides, *Mentha canadensis* L. polysaccharides, wound healing, skin defect model

## Abstract

Natural hydrogels are growing in interest as a priority for wound healing. Plant polysaccharides have a variety of biological pharmacological activities, and chitosan hydrogels have proven strong antimicrobial effects, but hydrogels prepared with polysaccharides alone have certain deficiencies. Polysaccharides from flowers of *Lonicera japonica* Thunb. (LP) and the aerial parts of *Mentha canadensis* L. (MP) were extracted and oxidized by sodium periodate (NaIO_4_) and then cross-linked with oxidized-carboxymethylated chitosan (O-CCS) to develop oxidized plant- polysaccharides-chitosan hydrogels (OPHs). SEM observation showed that OPHs had porous interior structures with interconnecting pores. The OPHs showed good swelling, water-retention ability, blood coagulation, cytocompatibility properties, and low cytotoxicity (classed as grade 1 according to United States Pharmacopoeia), which met the requirements for wound dressings. Then the cutaneous wound-healing effect was evaluated in BALB/C mice model, after 7 days treatment, the wound-closure rate of OPHs groups were all greater than 50%, and after 14 days, all were greater than 90%, while the value of the control group was only 72.6%. Of them, OPH-2 and OPH-3 were more favorable to the wound-healing process, as the promotion was more significant. The plant polysaccharides and CS-based hydrogel should be a candidate for cutaneous wound dressings.

## 1. Introduction

Skin has many functions for humans and animals, but when impaired, it can be a bacterial medium with a moist environment and adequate nutrients that promote infection [1], which will increase inflammation effusion and prevent wound healing [2]. According to the MarketsandMarkets report, the wound-care products market globally is projected to grow from USD 19.3 billion in 2021 to USD 27.8 billion by 2026, with a compound annual growth rate of 7.6% [3]. Therefore, wound-care products have attracted more attention among biomaterials’ applications [4], and the development of an ideal product with both hemostatic and anti-infection properties is necessary. In recent year reports, biomaterials for wound restoration have mainly included electrostatic spinning fibers, nanoparticles, biofilms, hydrogels, etc. [5,6,7,8,9]. Among them, hydrogels are growing in interest for their multifunction capabilities, extracellular matrix-like structure, and high moisture.

Hydrogels have reticular formation, are made from hydrophilic high molecular compound interactions via physical or chemical crosslinking, and have high permeability to oxygen, drug molecules, and other water-soluble substances. Hydrogels have another advantage of high ratio of drug loading; numerous studies have shown that hydrogels loading antimicrobial agents [10,11], cytokines [12,13,14], antioxidants [15], stem cells [16,17,18,19], or combined use of new technology [20,21] have very good application value in biomedical fields. Stimuli-responsiveness is an additional preponderance, and stimuli-responsive hydrogels have been successfully applied to biomedical applications, with use as drug-carriers, scaffold materials, sensors, actuators, etc. [22]. According to the source of raw material, hydrogels can be divided into natural and synthetic hydrogels [23]. Compared with synthetic polymers, natural polymers, such as animal and plant extraction with inherent multiple bioactivities, biological compatibility, and stability, can reduce cytotoxicity to tissue [24].

Natural polysaccharides are abundant, biocompatible, and biodegradable; have wide pharmacological activities; fit sustainable development; and have received much attention as candidates for hydrogel preparation [25]. Meanwhile, a huge number of hydroxyl, carboxyl, and amino groups on the saccharide units of polysaccharides provide opportunities for derivatization, which is not only conducive to format polysaccharide-based hydrogels but also directly affects cells behavior of organism. Chitosan (CS) is an abundant natural polysaccharide and has the characteristics of biocompatibility, biodegradation, antibacterial, etc., and it has also been prepared into various materials [26,27,28]. CS chains contain a large amount of hydroxyl and amino groups and can be modified physically and/or chemically to meet the actual needs.

Recently, hydrogel made by polysaccharides, such as cellulose, CS, hyaluronic acid, alginate, dextran, starch, chondroitin sulfate, and gellan gum, has shown great potential in biomedicine [29]. However, the obtained hydrogels prepared with these polysaccharides alone are uncontrollable in the pore size, mechanical strength, and swelling capacity [30,31] and have certain deficiencies, such as their inability inhibit inflammation, difficulty in regulating immunity, etc. [32]. Sulfated polysaccharide fucoida, extracted from brown algae, has been used to prepared alginate/chitosan/sulfated polysaccharide fucoida composite hydrogels, and the produced hydrogels exhibit high mechanical strength, excellent biocompatibility, and biodegradability [32].

Research found that natural polysaccharides can be recognized by the membrane receptors of macrophages and lymphocytes through their certain glycosyl units (e.g., mannose, β-glucan, etc.) [33]. These findings inspired Niu et al. use *Bletilla striata* polysaccharide to prepare a hydrogel as the cell scaffold; the results show that the collagen hydrogel coated with acetylated Bletilla polysaccharide efficiently activates tissue macrophages and improves the ability of tissue repair and regeneration [33]. Flowers of *Lonicera japonica* Thunb. as a traditional Chinese medicine named “Jin Yin Hua” possesses multiple bioactivities, such as anti-allergenic, anti-inflammatory, and neuroprotective effects [34]. *L. japonica* Thunb. polysaccharide (LP) is a major active ingredient of *L. japonica* Thunb. and is composed of glucose, mannose, arabionse, galactose, galacturonic acid, and rhamnose [34,35]; it also possesses antibacterial, anti-inflammatory, antioxidative, hypolipidemic, and anti-diabetic activity and inhibits cancer cells growth and reinforces immunity activity [36,37,38,39]. *M. canadensis* L. is known as “Bo He” in China and possesses anti-microbial, anti-inflammatory, anti-oxidant, anti-allergenic, anti-viral, and anti-tumor activities. Polysaccharides of *M. canadensis* L. (MP) are composed of glucose, mannose, rhamnose, gluconic acid, galactose, galacturonic acid, and arabionse [40,41]. They exhibit antioxidative, viral-inhibiting, and bacterial inflammatory activities [40,41,42]. LP and MP both contain the mannose and glucose units, and their activities satisfy the properties of wound dressing, and these two plants are cheap and suitable for public use.

In addition, up to now, few plant polysaccharide-based hydrogels have been studied, and especially, there are no LP/MP-based hydrogels referenced in the literature. Considering CS hydrogels have strong antimicrobial effects [24,43], a kind of natural composite hydrogels (OPHs) based on LP, MP, and CS were synthesized. Then, OPHs with various compositions (oxidized-plant polysaccharides to oxidized-carboxymethylated chitosan ratio) were analyzed in terms of morphological characteristics, gel content, swelling capacity, water-retention ability, rheological performance, blood compatibility, and cell compatibility to investigate whether the OPHs were suitable for wound dressings. Finally, the wound-healing effect was evaluated in BALB/C mice model; this study’s results will facilitate future investigations of plant-polysaccharide-based hydrogels as medical material.

## 2. Materials and Methods

### 2.1. Materials

Chitosan (deacetylation degree ≥ 90%, Mw = 205 kD) was purchased from Zhejiang Aoxing Biotechnology Co., Ltd., Zhejiang, China. Chloroacetic acid and NaIO_4_ were purchased from Tianjin Evergreen Chemical Reagent Manufacturing Co., Ltd., Tianjin, China. 4′,6-diamidino-2-phenylindole (DAPI), hematoxylin and eosin reagents, Bovine serum albumin (BSA) and fetal bovine serum (FBS) were obtained from Shanghai Aladdin Biochemical Technology Co., Ltd., Shanghai, China. Mouse fibroblasts cells (MFC L929) and BD vacutainer ACD tubes (364,606) were purchased from Chongqing Yes Service Biomedical Tech., Inc., Chongqing, China. Dulbecco’s modified eagle medium (DMEM) was purchased from Sigma−Aldrich Company (St Louis, MO, USA). Other chemical reagents used were of analytical grade and purchased from Sinopharm Chemical Reagent Co., Ltd., Beijing, China.

Freeze-dryer (SCIENIZ-18N) was from Ningbo Biotechnology Co. Ltd., Ningbo, China. FT-IR spectrometer (Nicolet 170SX) and rotated rheometer (RS 6000) were from Thermo Fisher Scientific Inc., Waltham, MA, USA. Scanning electron microscope (Jeol JSM-6010LV) was from JEOL, Tokyo, Japan. Magnetic stirring (78HW-1) was form Jiangsu Zhengji Instrument Co., Ltd., Jiangsu, China, centrifugation (TD5A-WS) form Changsha Xiangyi Centrifuge Instrument Co., Ltd., Hunan, China and water bath (HH-4) from Beijing Kowei Yongxing Instrument Co. Ltd., Beijing, China. Fluorescence microscope (UY203i) was from Beijing Rong xing guang heng technology Co. Ltd., Beijing, China.

*L. japonica* Thunb. and *M. canadensis* L. were purchased from local supermarket in Lanzhou City, Gansu Province, China, and identified by Prof. Zhang Weijie as the flower bud of *L. japonica* Thunb. and the aerial parts of *M. haplocalyx* Briq., respectively, according to the Pharmacopoeia of the People’s Republic of China 2020.

Male, 8-week-old BALB/C mice were obtained from Lanzhou Institute of Veterinary Medicine, Chinese Academy of Agricultural Sciences (Lanzhou, China).

### 2.2. Methods

#### 2.2.1. Preparation LP and MP

Flower bud of *L. japonica* Thunb. and the aerial parts of *M. haplocalyx* Briq. were dried at 50 °C for 24 h, crushed, and passed through a 60 mesh sieve; then, they were degreased with absolute alcohol twice. A total of 500 g of the degreased material was separately extracted with 15 L distilled water at 100 °C for 4 h, two times. The filtrates were concentrated to 1/10 of original volume, added with absolute ethyl alcohol to the concentrations until final concentrations increased up to 80%, and precipitated at 4 °C for 36 h. The precipitates were collected and washed with 95% ethanol, then deproteinized repeatedly by Sevage method (v (chloroform): v (n-butyl alcohol) = 4:1) [44,45]. The deproteinized fraction was lyophilized at −20 °C and named LP and MP, respectively. The polysaccharide content was quantified by Equation (1).

(1) $$\mathrm{polysaccha ride content}\;(\%)=\frac{C\times V\times N}{W\times 10^{6}}\times 100$$

Note: C is the polysaccharide concentration (mg/mL) calculated by the calibrated regression equation, N is the dilution multiple, V is the extraction volume (mL), and W is the mass of *L. japonica* and *M. canadensis* (g).

#### 2.2.2. Preparation of OPHs

Preparation of O-CCS: 10 g CS and 13.5 g NaOH was suspended into 100 mL isopropanol solution (w_isopropano__l_:w_water_ = 4:1) with continued whisking for 1 h at room temperature. A total of 15 g monochloroacetic acid was mixed in 20 mL isopropyl alcohol and then added to the CS alkaline solution dropwise. After reacting at 40 °C for 4 h under constant stirring, 40 mL 70% ethanol was added to stop the reaction. The solid was collected by filter and subsequently rinsed with 70%, 80%, and 90% ethanol to desalt and dewater; then, the obtained Na-O-CCS was dissolved in 100 mL 80% ethanol, added with 10 mL 37% hydrochloric acid, and stirred at 300 rpm for 30 min to desalination; the reaction mixture was then vacuum filtered, and the solid product was dialyzed with water for 36 h. The dialysis were precipitated with 3 times absolute ethanol at 4 °C for 14 h, and the solid was collected by suction filter and dried at 60 °C [46,47].

Preparation of OLMP: The weight ratio of LP and MP was determined as 3:1 according to the first stage of our research, with the actual oxidation degree, swelling ratio, and porosity as evaluation indexes. Firstly, LP and MP (3:1) were mixed together, and 4 g mixture was dissolved in 100 mL 50% ethanol with continuous whisking of 200 rpm. 6 g NaIO_4_ was dissolved in 100 mL distilled water and then dispersed into the polysaccharide solution with continuous stirring at 300 rpm for 8 h in darkness. Subsequently, ethylene glycol with an equimolar amount of NaIO_4_ was added into reaction system; 5 min later, 4 g NaCl was added also and stirred for 15 min to quench the reaction. After filtration, the solid was dispersed in deionized water and dialyzed. The dialyses were precipitated with 3 times the volume of absolute ethanol at 4 °C for 16 h, and then, the solids were collected by suction filter, vacuum-dried, and named OLMP [48]. The oxidization degree of OLMP was measured by using an element analyzer (Flash EA-1112).

**Figure 1:**
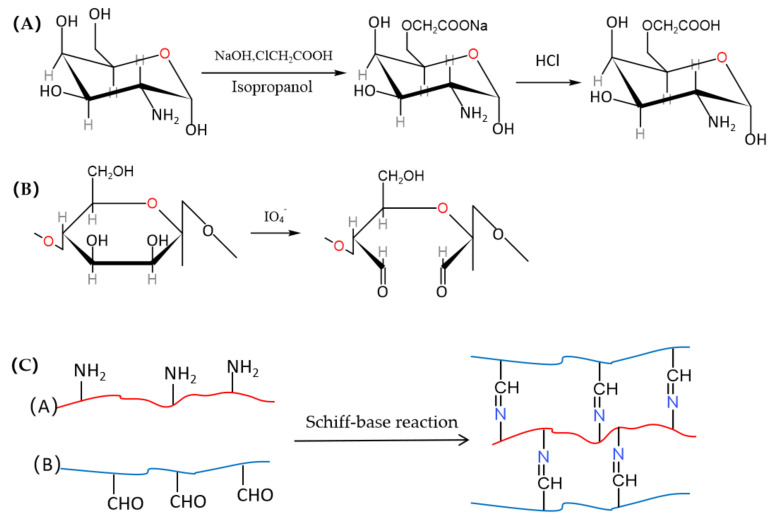
The schematic of O-CCS (**A**), OP (**B**), and OPHs (**C**), respectively.

Preparation of OPHs: 4% OLMP solution and 4%, 8%, and 16% O-CCS solution (4% for OPH-1, 8% for OPH-2, and 16% for OPH-3) were prepared using boiled distilled water under constant stirring with 200 rpm. After dissolving and cooling, OLMP and O-CCS solutions (*v*:*v* = 1:1) were mixed together in glass tubes, stirred at 100 rpm for 10 min, and then stood at 37 °C for 10 h to form hydrogels (OPHs); then, prepared OPHs were kept below 4 °C for later usage. The schematic is shown in Figure 1.

#### 2.2.3. Scanning Electron Microscopy (SEM) and Structural Characteristics of OPHs

The hydrogels were lyophilized at −60 °C for 24 h; then, transverse sections were cut by a cold knife, fixed with conductive tape, and gilt at 40 mA for 20 min; then, the surface and cutting plane were observed with a SEM at 20 kV.

To compare the changes of functional groups after crosslinking, the OLP, OMP, O-CSS, and OPH samples were lyophilized, and the FT-IR spectra were measured over the range from 400–4000 cm^−1^ using FT-IR spectrometer.

#### 2.2.4. Rheological Analysis

Rheological measurement of OPHs was performed using a rheometer. OPHs was loaded onto the platform, and dynamic frequency sweeps from 0 to 10 Hz were performed at constant strain rate 0.5%, 25 °C to determine the storage modulus (G′) and loss modulus (G″) [49,50].

#### 2.2.5. Gel Content

Gel content is an indicator of the cross-linking degree. To evaluate the gel content of OPHs, they were dried at 50 °C until constant weight (*m*_2_) and then separately extracted in distilled water at 100 °C for 48 h; then, they were dried again at 50 °C to constant mass (*m*_1_). The gel content was calculated as Formula (2) [51,52]:

(2) $$\mathrm{Gel content}(\%)=\frac{m_{1}}{m_{2}}\times 100$$

#### 2.2.6. Swelling and Water-Retention Characteristics

Completely dried and pre-weighed OPHs were separately immersed in 50 mL buffer solution (pH = 6.8) at 37 ± 0.5 °C. At certain time intervals up to the existence of equilibrium, the OPHs were taken out, and absorbent paper was applied to quickly remove the excess solution on the surface. Subsequently, they were weighted with an electronic balance [53]. Then, the swollen OPHs were set in Petri dishes at room temperature, 50% relative humidity, and weighted at certain time intervals until weight stabilized [54]. The swelling ratio and water retention were calculated based on Formulas (3) and (4):

(3) $$\mathrm{Swelling rate}\;(\%)=\left(\frac{m_{t}-m_{d}}{m_{d}}\right)\times 100\%$$

(4) $$\mathrm{Water retention}\;(\%)=\left(\frac{m_{i}-m_{d}}{m_{t}-m_{d}}\right)\times 100\%$$

Note: *m_d_* was the dried OPHs weight, and *m_t_* and *m_i_* were the mass of OPHs weighted in swelling stage and water evaporation stage at the time of intervals, respectively.

#### 2.2.7. In Vitro Blood Coagulation Properties

Blood coagulation was evaluated by the blood-clotting study and reflected by blood-clotting index (BCI) [55]. A total of 10 mg OPH-1, OPH-2, and OPH-3 were separately put into conical flasks and immersed in a constant-temperature water bath at 37 °C for 5 min. Then, 0.27 mL of each blood sample (ACD whole blood containing 8% (*v*/*v*) 0.2 mol/L CaCl_2_) was carefully seeded on the OPHs surface. After incubating for 10 min, 10 mL deionized water was slowly dripped along the wall of the conical flasks to avoid disturbing the blood. Then, 10 mL solutions were siphoned out from the conical flasks and centrifuged (1500 rpm for 1 min). The supernatant was slowly poured into test-tubes with 40 mL deionized water and insulated in 37 °C for 60 min. Subsequently, the absorbances were measured at 542 nm. BCI was calculated according to Formula (5), and 0.27 mL ACD whole blood mixed in 10 mL deionized water as control [52].

(5) $$\mathrm{BCI}\;(\%)=\frac{A_{s}}{A_{0}}\times 100$$

Note: *A_s_* is absorbance of blood in contact with OPHs sample; *A*_0_ is absorbance of ACD whole blood in water.

#### 2.2.8. Cytotoxicity and Cytocompatibility Test

The cytotoxicity of OPHs was assessed by MTT assay method [56]. The lyophilized hydrogels were sliced, sterilized in 75% alcohol, and rinsed with sterilized PBS; subsequently, OPHs were separately immersed in DMEM and placed in constant temperature incubator shaker with 48 h oscillation at 37 °C to obtain the extraction. MFC (1 × 10^4^ cells/well) were seeded in the 96-well plates with DMEM medium (containing 10% FBS) and incubated at 37 °C for 24 h; when the cell was spread over the bottom of the well, the DMEM of sample groups was replaced with 100 μL OPH extraction, and that of the control group was replaced with fresh medium. After 24 h culturing, each 200 μL of MTT (0.5 mg/mL in PBS) was added to the wells and stood for 4 h. Then, the supernatant in the wells was pipetted out carefully, and 150 μL DMSO was added in each well. The absorbance values were measured at 570 nm, and the cytotoxicities of OPHs were displayed by the relative growth rate (RGR) using Formula (6):

(6) $$RGR\;(\%)=\frac{As}{Ac}\times 100$$

Note: *A_s_* is absorbance of co-culture sample, and *A_c_* is absorbance of control group.

The cytocompatibility of OPHs were evaluated by material and cell co-culture [27]. MFC were seeded in DMEM (containing 10% FBS and 10% DAPI) at 37 °C for 24 h and then rinsed with sterile PBS at least 6 times to wash off the uncombined DAPI. Then, the cells were digested by trypticase, collected by centrifugation, and cell suspension was prepared using DMEM (containing 10% FBS). The sterilized hydrogel slices were placed in 24-well plates, and cell suspension was added in; after 24 h co-culture, the cellular adherence and growth were observed with fluorescence microscope.

#### 2.2.9. In Vivo Skin Wound Healing

A mice model was used to evaluate the wound-healing effect of OPHs. A total of 40 BALB/C mice, weighted about 25 g, were anesthetized by intramuscular injection 4% chloral hydrate (0.01 mL/g). After the back hair was shaved with a shaving machine and removed with depilatory cream, the skin was disinfected with 75% ethanol, and a full-thickness skin wound (2 mm deep) with a diameter of 6 mm was made using a circular aseptic skin punch of each mouse. Then, 40 mice were randomly divided into four groups equally (OPH-1, OPH-2, OPH-3, and control group). The wounds of three experimental groups were immediately covered with OPH-1, OPH-2, and OPH-3, and that of the control group were cleaned with amounts of saline solution and then covered with asepsis dried gauze. Then, all wounds of the four groups were fixed with an elastic adhesive bandage. All mice were kept in individual cages; sufficient food and water were provided ad libitum [57].

After 7 days treatment, wounds were examined and photographed using the Toupview image analysis software after calibration; then, OPHs were changed, or the wound was cleaned with amounts of saline solution depending on the group, and wound closure rate was calculated using Equation (7) [58]:

(7) $$\mathrm{Wound closure rate}\;(\%)=\frac{A_{0}-A_{t}}{A_{0}}\times 100$$

Note: *A*_0_ is the initial trauma area; *A_t_* is trauma area measured at 7th or 14th day.

After 14 days treatment, wounds also were examined and photographed; then, mice were euthanized by injecting excess dose of chloral hydrate and then executed by dislocation. To investigate the influence of OPHs to wound tissue, the entire wound with adjacent normal skin tissue, including dermis and subcutaneous tissue, was excised and fixed in formalin for 48 h, processed by dehydration, embedded in paraffin, and then sliced to 3–5 mm thick. The slices were then stained with hematoxylin and eosin (HE) reagent and observed by image analysis software (Toupview). The blood was collected by BD vacutainer^®^ ACD tubes and preserved.

## 3. Results

### 3.1. Preparation of MP and LP

In total, 180 g LP and 53.78 g MP were obtained, and the polysaccharide content was 62.47 ± 3.02% and 57.80 ± 2.610% for LP and MP, respectively. The DCA of O-CCS was 41.26 ± 2.14%, and the actual aldehyde content of OLMP was revealed as oxidation of 63.0%.

### 3.2. Performance Assessment of OPHs

#### 3.2.1. Morphology and FTIR Analysis

**Figure 2:**
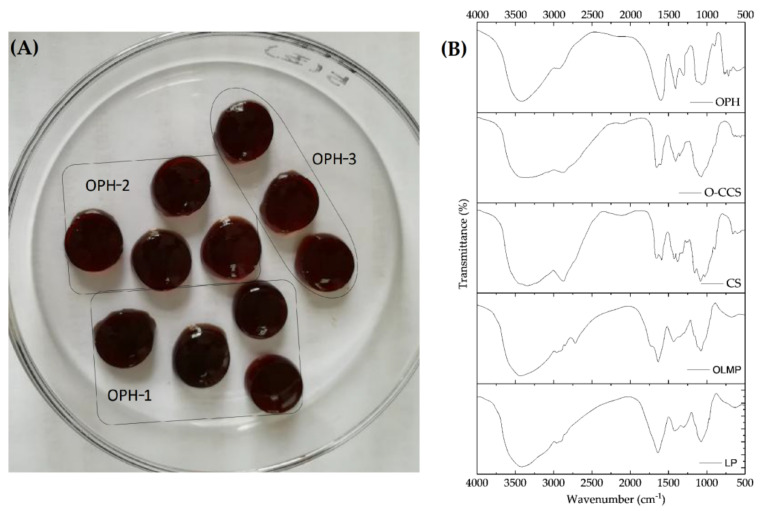
Performance of OPHs (**A**) and FTIR characteristic of OLMP, O−CCS, and OPH−1 (**B**).

As Figure 2A displayed, the morphology of OPHs was clear and uniform and showed no difference among OPH-1, OPH-2, and OPH-3 to the naked eye.

Figure 2B showed the FTIR characteristic of OLMP, CS, O-CSS, and OPH-1. The FTIR spectrum of CS exhibited two peaks at 1657 and 1598 cm^−1^, which could be assigned to C=O and N-H vibrations of amide [32] The O-CCS has two new absorption peaks at 1407 cm^−1^ and 1587 cm^−1^, which could belong to the symmetric and asymmetric stretch vibrations of COO- [46]. Compared to CS, the C-O stretching band at 1030 cm^−1^, corresponding to the OH shifted to 1068 cm^−1^, corresponded to C-O-C stretching, illustrating a carboxymethylation had occurred at C_6_-OH [46]. OLMP showed two peaks at 1730 and 2720 cm^−1^, which could be assigned to the aldehyde groups [57]. In OPH spectra, the -CHO band of OLMP at 1730 cm^−1^ disappeared; meanwhile, the amide peaks of O-CCS transformed to a single, sharp absorption peak at 1635 cm^−1^, which could be ascribed to the formation of a C=N structure among the amino groups of O-CCS and the aldehyde group of OLMP [57].

#### 3.2.2. SEM of OPHs

**Figure 3:**
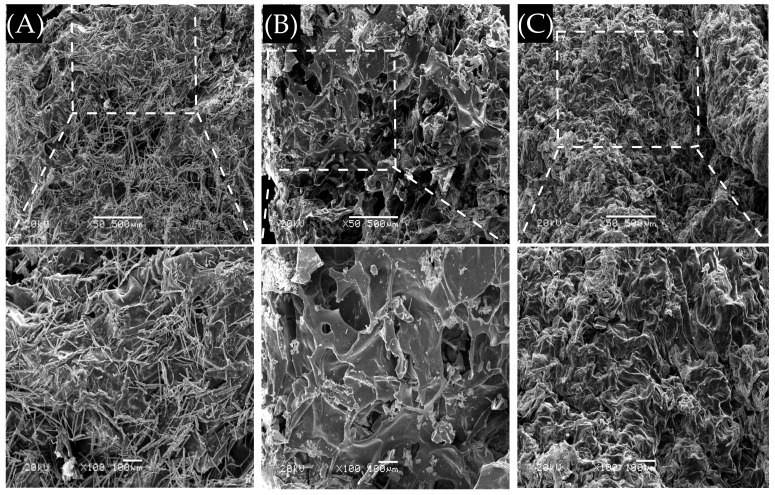
SEM images of the OPHs. (**A**–**C**) OPH-1, OPH-2, and OPH-3, respectively. They were magnified as multiples of 50 and 100, and the scales are 500 µm and 100 µm, respectively.

As exhibited in Figure 3, OPHs had porous interior structures with interconnecting pores; as the proportion of OLMP to O-CCS changed from 1:1 to 1:3, more crosslink formed, and OPH-3 possessed a tighter and more homogeneous morphology, while OPH-1 was loose and heterogeneous.

#### 3.2.3. Rheological Analysis and Gel Content

**Figure 4:**
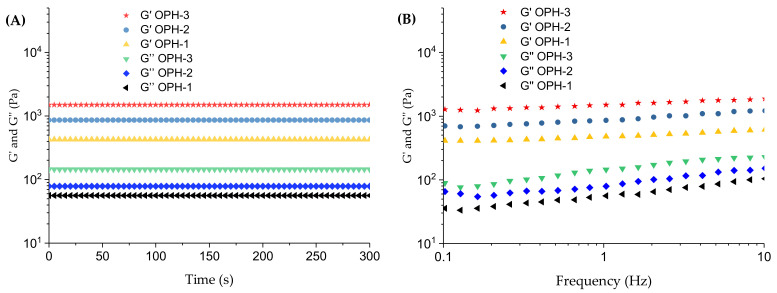
G′ and G″ of OPHs with the increase of time (**A**) and dynamic frequency from 0.1 to 10 Hz (**B**).

Hydrogels have the double advantage of being both a viscous liquid and elastic solid, represented as storage modulus (G′) and loss modulus (G″). As exhibited in Figure 4, G′ and G″ of the samples did not change significantly with the increase of dynamic time or frequency. Moreover, the G′ values were always greater than the G″ values, and G′ and G″ values both increased with the increase of O-CCS concentration. The quantitative analyses of cross-linking degree were carried out on the basis of the gel content; the value of OPH-1, OPH-2, and OPH-3 was between 66.75 ± 3.54%, 83.33 ± 6.22%, and 84.44 ± 5.10%, respectively.

#### 3.2.4. Swelling and Water Retention

**Figure 5:**
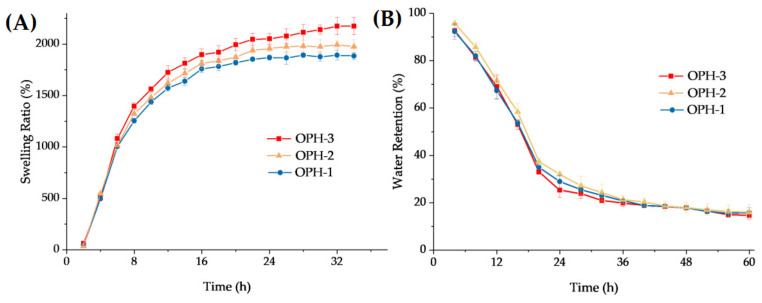
Swelling ratio (**A**) and water-retention ratio (**B**) of OPHs (mean ± SD, *n* = 3).

The swelling ratio can be used to reflect the swelling behavior [59], and as exhibited in Figure 5A, the swelling radio increased fast in the first 12 h and then gradually slowed down until reaching swelling equilibrium. The equilibrium of OPH-3 and OPH-2 was reached at 32 h, with the highest swelling ratio of 2175.5 ± 66.1% and 1883.2 ± 35.3%, respectively. OPH-1 reached equilibrium at 24 h, the swelling ratio of 1757.7 ± 86.8%. The water-retention ratio is shown in Figure 5B, where it can be seen that the water was quickly lost in 24 h and then slowed down after. After 60 h, OPH-1, OPH-2, and OPH-3 still retained 10% to 20% water.

### 3.3. In Vitro Blood Coagulation Properties, Cytotoxicity, and Compatibility of the OPHs

**Figure 6:**
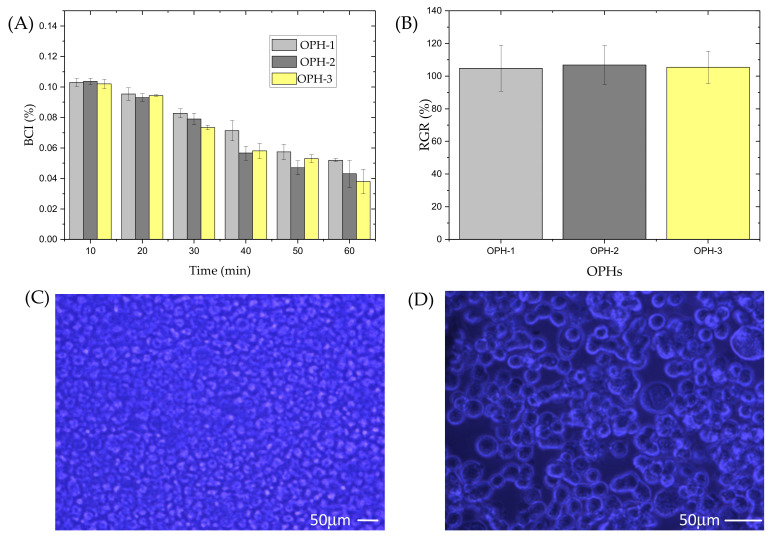
OPH samples blood coagulation performance, cell compatibility, and cytotoxicity. (**A**) BCI and (**B**) RGR of OPH-1, OPH-2, and OPH-3 (mean ± SD, *n* = 6). (**C**) Cell proliferation on the surface of OPH-3. (**D**) DAPI fluorescent staining of MFC.

As shown in Figure 6A, the BCI decreased with time, and at 60 min, the value reached 0.052 ± 0.001%, 0.043 ± 0.012%, and 0.038 ± 0.008% for OPH-1, OPH-2, and OPH-3, respectively. It can be observed from Figure 6B that for MFC cultured on OPHs, the RGR was 104.80%, 106.87%, and 105.42% for OPH-1, OPH-2, and OPH-3, respectively.

The biocompatibilities was evaluated by observing the growth status of cells after co-culturing with OPHs. The proliferation ability of MFC on the surface of OPH-3 is shown in Figure 6C,D, and it can be observed that the cells proliferated normally and spread well.

### 3.4. In Vivo Skin Wound Healing and Histocompatibility Analysis

**Figure 7:**
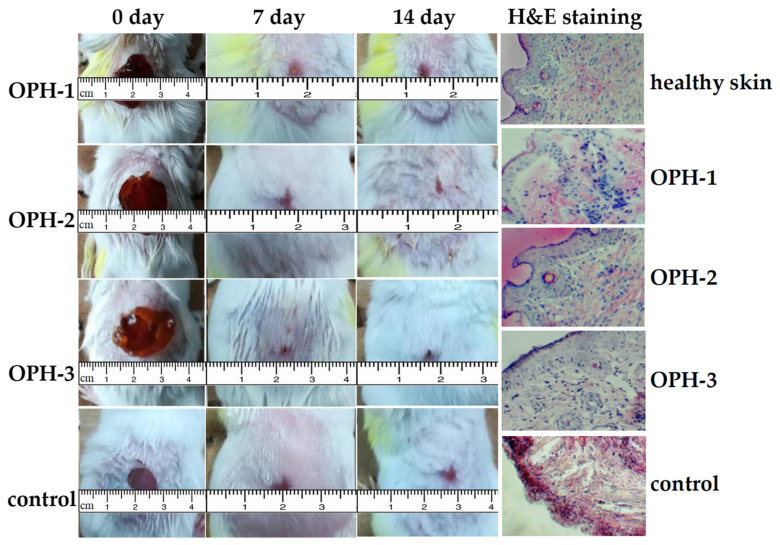
Photographs of wound healing and H&E staining for healthy skin, OPHs, and control (100×).

The wound-closure rates of the OPHs and control groups are listed in Table 1. The photographs are shown in Figure 7. After 7 days treatment, the wound-closure rate of OPHs groups were all greater than 50%, and after 14 days, they were all greater than 90%, while the value of control group was only 72.6%. As H&E staining results showed, healthy skin has a continuous dermis and epidermis, sparse collagen tract, and many hair follicles arranged under dermis. After 14 days, the control group showed a discontinuous epidermis and severe inflammation with the absence of hair follicles, indicating that a scar is developing. The increase of collagen bundles in the dermis and the regeneration of epidermis without inflammation and scars is essential for wound healing [56]. After 14-day treatment of OPHs, the OPH-2 and OPH-3 groups also showed continuous epidermis and organized collagen bundles, with the collagen cells arranged neatly and regularly. The OPH-1 showed a discontinuous epithelium but loosely arranged collagen bundles, and the inflammation was obviously less than the control group.

## 4. Discussion

Hydrogels have swelling ability, softness, and water-retention features and thus can absorb blood and wound exudates and provide a soft and humid environment. This is a unique characteristic in the field of wound repair [60], and especially, the natural hydrogels have priority for their potential nontoxicity [61].

CS as the only cationic natural polysaccharide is not only nontoxic in both animal models and humans but also shows good antimicrobial properties and thus has been widely prepared into various materials [31]. However, the application of CS in wound dressing is limited for its low solubility in neutral water [46]. In recent years, a series of soluble derivatives of CS have been prepared, such as O-CCS, which was prepared by covalent attachment of hydroxyl group on the C6-OH; in the presence of the new carboxylion, the protonation of the amine group in the C-2 position was enhanced, so the solubility of O-CCS in a wide pH range increased, and its application as an antimicrobial agent is also much broader than CS [62]. In order to enrich the activity, it is necessary to incorporate more raw bioactivities into it [2]. Plant polysaccharides are abundant resources and have wide pharmacological activities, including an inhibiting effect on bacteria and viruses.

In this work, OPHs were fabricated via the regioselective oxidation of LP and MP with NaIO_4_ and then crosslinked with O-CCS by Schiff-base linkages between the aldehyde of oxidized polysaccharides and imine groups of O-CCS. The whole production process of OPHs required no harsh conditions, which could be one of the major advantages.

Ideal wound dressings should have good elasticity and adhesion. The elasticity property could prevent the wound dressings from deformation or damage under the action of external forces, and adhesion is helpful to control or stop bleeding [29]; meanwhile, cells are sensitive to their surroundings, including the viscoelasticity of the matrices [63]. The G′ values of prepared OPHs were higher than the G″ values, showing typical elastic solid properties [50]. Furthermore, G′ and G″ did not change significantly with the increase of low dynamic frequency or time, and their independence proved the covalent nature of the hydrogel matrices [63] and the stability [64]. G′ and G″ values also both increased with the increase in O-CCS concentration, and this might be because cross-linked groups on OLMP backbone increased with the rise in O-CCS content [65], which was consistent with the gel content measurement results. The gel content was increased with the increase in material ratio of O-CSS, which corresponds to the SEM results, indicating that the cross-link increases with increasing O-CCS content.

Hydrogels display excellent swelling capacity and water retention due to their porous structure, crosslinked networks, and hydrophilic groups. Swelling capacity mainly depends on the hydrophilic groups number and the internal network structure [56]. OPHs swelled quickly in PBS at the original 16 h and then expanded slowly until equilibrium with the swelling ratio reached more than 1800%. Under standing room temperature and 50% relative humidity conditions for 60 h, the OPHs still retained 10% to 20% water owing to the porous structure, and the intermolecular hydrogen bonds formation between the water molecules and the hydrogel accelerates the absorption and blocks the evaporation of water molecules [49]. That means the swelling capacity and water retention of OPHs were excellent.

Wound dressings should possess a superior blood-clotting function, and the quantitative value of BCI is lower, suggesting the material possesses a certain extent of coagulation effect. At 60 min, the lowest BCI demonstrated by OPH-3 reached 0.038. Low cytotoxicity and good cyto-compatibility are also prerequisites for wound dressings. The cytotoxicity of OPHs were detected by MTT assay: the higher the RGR value, the lower the cytotoxicity of the OPHs. The RGR values of OPHs were all greater than 100%, and the cell toxicity can be classed as grade 0 according to United States Pharmacopoeia, which indicated that OPH-1, OPH-2, and OPH-3 all had low cytotoxicity. Meanwhile, OPHs exhibited good cyto-compatibility. All these properties suggest that OPHs samples are fit as wound dressings. Hence, the skin wound-healing effect was researched by building a trauma model in mice, and after 14-day treatment, the wound-closure rate of the three OPH groups were all greater than 90%, while the value of the control group was only 72.6%. Of the OPH groups, the wound-closure rate of OPH-3 was highest. The histological examination results also demonstrated the OPHs helped to heal skin wounds, especially OPH-2 and OPH-3. The cytocompatibility of OPHs provides a suitable setting for fibroblast proliferation and thus the continuous formation of epidermis and collagen fibers in the dermis. Research shows that CS molecules can facilitate cell proliferation and growth and organize collagen deposition [56]. Additionally, the antibacterial, anti-inflammatory, etc., activities of LP and MP also play a crucial role in wound healing. All these results demonstrate that plant polysaccharides and CS-based hydrogel offer unique structural and functional characteristics that make this material suitable as a candidate for wound dressings.

## Figures and Tables

**Table 1:** Wound-closure rate at 7th and 14th day (mean ± SD, *n* = 10).

Days of Observation	Wound-Closure Rate (%)			
	OPH-1	OPH-2	OPH-3	Control
7 days	54.4 ± 8.4	57.3 ± 7.2	60.1 ± 9.1	48.6 ± 6.2
14 days	90.6 ± 6.9	93.5 ± 8.1	95.1 ± 8.7	72.6 ± 8.1

## Data Availability

The data presented in this study are available on request from the author (e-mail: xuehongyan926@163.com).
